# Are Baseline Levels of Gas6 and Soluble Mer Predictors of Mortality and Organ Damage in Patients with Sepsis? The Need-Speed Trial Database

**DOI:** 10.3390/biomedicines10020198

**Published:** 2022-01-18

**Authors:** Francesco Gavelli, Luca Molinari, Marco Baldrighi, Livia Salmi, Filippo Mearelli, Nicola Fiotti, Filippo Patrucco, Chiara Airoldi, Mattia Bellan, Pier Paolo Sainaghi, Salvatore Di Somma, Enrico Lupia, Efrem Colonetti, Maria Lorenza Muiesan, Gianni Biolo, Gian Carlo Avanzi, Luigi Mario Castello

**Affiliations:** 1 Department of Translational Medicine, Università del Piemonte Orientale, 28100 Novara, Italy; francesco.gavelli@uniupo.it (F.G.); marco.baldrighi590@gmail.com (M.B.); salmi.livia@gmail.com (L.S.); filippo_patrucco@hotmail.it (F.P.); chiara.airoldi@uniupo.it (C.A.); mattia.bellan@med.uniupo.it (M.B.); pierpaolo.sainaghi@med.uniupo.it (P.P.S.); giancarlo.avanzi@uniupo.it (G.C.A.); luigi.castello@med.uniupo.it (L.M.C.); 2 Emergency Medicine Department, Azienda Ospedaliero-Universitaria “Maggiore della Carità”, 28100 Novara, Italy; 3 Unit of Internal Medicine, Department of Medicine, Surgery and Health Sciences, University of Trieste, 34129 Trieste, Italy; filippome@libero.it (F.M.); fiotti@units.it (N.F.); biolo@units.it (G.B.); 4 Unit of Emergency Medicine, Department of Medical Surgical Sciences and Translational Medicine, University “Sapienza” of Rome, 00189 Roma, Italy; salvatore.disomma@uniroma1.it; 5 Unit of Emergency Medicine, Department of Medical Sciences, University of Turin, 10126 Torino, Italy; enrico.lupia@unito.it; 6 Unit of Internal Medicine, Department of Clinical and Experimental Sciences, University of Brescia, 25121 Brescia, Italy; colonetti@gmail.com (E.C.); marialorenza.muiesan@unibs.it (M.L.M.); 7 SEUM 118, Azienda Ospedaliera Universitaria Integrata di Verona, 37126 Verona, Italy; 8 Unit of Internal Medicine, Azienda Ospedaliera SS. Antonio e Biagio e Cesare Arrigo, 15121 Alessandria, Italy

**Keywords:** Gas6, sMer, biomarkers, TAM receptors, sepsis, coagulopathy, acute kidney injury

## Abstract

Soluble tyrosine kinase receptor Mer (sMer) and its ligand Growth arrest-specific protein 6 (Gas6) are predictors of mortality in patients with sepsis. Our aim is to clarify whether their measurement at emergency department (ED) presentation is useful in risk stratification. We re-analyzed data from the Need-Speed trial, evaluating mortality and the presence of organ damage according to baseline levels of sMer and Gas6. 890 patients were eligible; no association with 7- and 30-day mortality was observed for both biomarkers (*p* > 0.05). sMer and Gas6 levels were significantly higher in acute kidney injury (AKI) patients compared to non-AKI ones (9.8 [4.1–17.8] vs. 7.9 [3.8–12.9] ng/mL and 34.8 [26.4–47.5] vs. 29.8 [22.1–41.6] ng/mL, respectively, for sMer and Gas6), and Gas6 also emerged as an independent AKI predictor (odds ratio (OR) 1.01 [1.00–1.02]). Both sMer and Gas6 independently predicted thrombocytopenia in sepsis patients not treated with anticoagulants (OR 1.01 [1.00–1.02] and 1.04 [1.02–1.06], respectively). Moreover, sMer was an independent predictor of both prothrombin time-international normalized ratio (PT-INR) > 1.4 (OR 1.03 [1.00–1.05]) and sepsis-induced coagulopathy (SIC) (OR 1.05 [1.02–1.07]). An early measurement of the sMer and Gas6 plasma concentration could not predict mortality. However, the biomarkers were associated with AKI, thrombocytopenia, PT-INR derangement and SIC, suggesting a role in predicting sepsis-related organ damage.

## 1. Introduction

Sepsis is a life-threatening organ dysfunction caused by a dysregulated host-response to infection that affects about 31.5 million people every year; the in-hospital mortality ranges between 17% and 26% [1]. Both a prompt diagnosis and prognostic assessment are necessary when sepsis is suspected in the Emergency Department (ED). An early diagnosis allows clinicians to select patients who benefit from immediate antibiotic treatment [2]. On the other hand, risk stratification permits one to identify septic patients at a higher risk of mortality or severe organ damage, who may need early intensive care unit (ICU) admission [3,4].

To improve both the accuracy and speed of the diagnostic process, as well as the early prognostic stratification of septic patients in the ED, many biomarkers, such as c-reactive protein, procalcitonin and lactate, have been proposed. Nonetheless, c-reactive protein is a very nonspecific marker of inflammation, and it is not useful for the diagnosis of sepsis when compared to procalcitonin [5,6]. However, despite the increasing diffusion in clinical practice observed in the last few years, current guidelines are against the use of procalcitonin when deciding whether to start antimicrobials [7]. The elevation of the plasma lactate concentration and its variation in time are widely used in the prognostic evaluation of septic patients [8,9,10,11,12], but doubts remain about their interpretation, due to the lack of specificity [13]. Thus, other biomarkers have been tested, alone or in combination [14,15,16,17,18]; however, the ideal biomarker is still lacking.

Recently, the role of the tyrosine kinase receptor Mer and its ligand growth arrest-specific 6 protein (Gas6) [19] has emerged as an important contributor to the inflammatory response in patients with sepsis, especially in the ICU. It has been shown that both a persistent overexpression of Mer and increased levels of Gas6 are related to an increased mortality rate in the ICU [20,21]. Furthermore, they have been shown to correlate to the development and the severity of sepsis-related organ dysfunction [22,23].

Nevertheless, no study has evaluated the role of these biomarkers as early predictors of mortality and organ damage in patients with sepsis. The aim of our study is to perform a secondary analysis of the Need-Speed trial [17] to evaluate the potential role of both Gas6 and the soluble form of Mer (sMer) as biomarkers for predicting mortality and organ damage in septic patients admitted to the ED.

## 2. Materials and Methods

### 2.1. Patients

We performed a secondary analysis of existing data from the Need-Speed trial. The trial design, methods and main results have been extensively described elsewhere [17]. Briefly, the Need-Speed trial was an observational multicenter study, enrolling consecutive adult patients admitted to five Italian Eds between March 2013 and March 2015. Patients were enrolled within 24 h of admission if they met two or more criteria of systemic inflammatory response syndrome (SIRS) [24]. The aim of this secondary analysis was to evaluate the prognostic value of Gas6 and sMer plasma concentrations in patients with sepsis at the ED presentation, defined according to the presence both of SIRS [24] and clinical or microbiologic signs of infection, in terms of mortality and organ damage development. The study was approved by the local ethical committee of each center involved and was conducted in conformity to the principles of the Declaration of Helsinki. Patients were prospectively and consecutively included.

### 2.2. Data and Samples Collection

At the time of enrolment, after informed consent was acquired, demographic, clinical and laboratory data were collected. At the same time, arterial and peripheral venous blood samples were drawn, urine samples were collected, and patients underwent a chest X-ray, according to the clinical judgement of the treating physician. Blood samples for biomarkers analysis were collected and centrifuged within 24 h of ED admission [25]. Gas6 was measured with a sandwich enzyme-linked immunosorbent assay (ELISA) developed and validated in our laboratory [26], while sMer was performed with a commercial ELISA kit. More details are provided in the Supplementary Material.

### 2.3. Mortality and Organ Damage

The 7- and 30-day mortality from enrolment was evaluated through telephone follow-up calls at 30 days, indicating whether the patient was alive or dead and the possible death date. The presence of concurrent organ damage was investigated in terms of acute kidney injury (AKI), coagulopathy (in terms of thrombocytopenia, prothrombin time-international normalized ratio (PT-INR) alteration and sepsis-induced coagulopathy (SIC)) and respiratory tract infection (RTI-r sepsis). The assessment is detailed in the Supplementary Material and Table S1.

### 2.4. Statistical Analysis

The normality of the data distribution was assessed through the Kolmogorov–Smirnov normality test. Data are expressed as the median [interquartile range] for continuous variables and as absolute numbers (percentages) for categorical variables. A comparison between groups was performed through the Mann–Whitney U test for continuous variables and through the Chi-square test for categorical variables.

Variables that were found to be with *p* < 0.05 at the univariate analysis were entered into a Cox proportional-hazards regression model (for 30-day mortality) or into a stepwise logistical regression model (for AKI, thrombocytopenia, PT-INR alteration, SIC, RTI-r sepsis); clinical relevance was also considered to identify covariates as candidate predictors for the multivariable model. The statistical significance was set at two-tailed *p* < 0.05. The statistical analysis was performed using MedCalc Statistical Software version 18.11.3 (MedCalc Software bvba, Ostend, Belgium; http://www.medcalc.org, accessed on 1 December 2021)).

## 3. Results

### 3.1. Patient Characteristics

Among the 1132 patients included in the primary analysis of the Need-Speed trial [17], 890 patients with a definitive diagnosis of sepsis were included in our analysis (Figure S1). The median age was 80 [72–87] years, 477 patients were males and 413 were females. The main baseline characteristics of our cohort are presented in Table 1. Gas6 was measured in 864 patients (97%) and sMer in 865 patients (96%), and their median plasmatic levels were 31.1 [23.2–43.5] ng/mL and 8.3 [4.0–14.4] ng/mL, respectively.

### 3.2. Mortality

**Figure 1:**
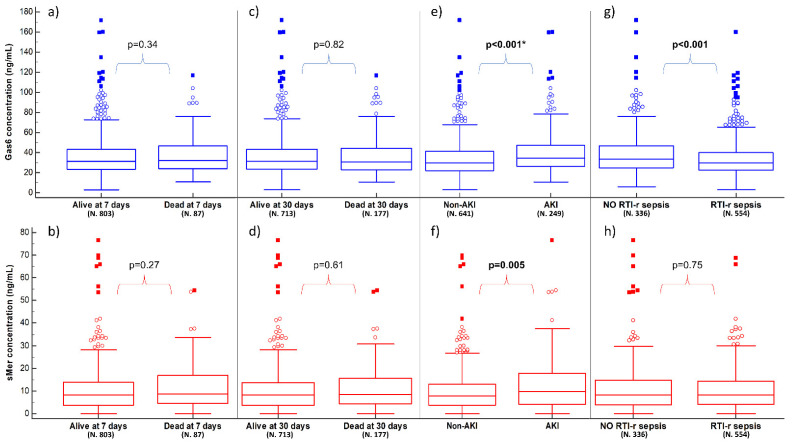
Plasma concentrations of Gas6 (blue boxes) and sMer (red boxes) according to 7- and 30-day mortality, AKI and RTI-r sepsis. Panels (**a**,**b**) show the results in relation to being alive or dead at 7 days; panels (**c**,**d**) show the results in relation to being alive or dead at 30 days; panels (**e**,**f**) show the results in relation to having AKI or not; panels (**g**,**h**) show the results in relation to having RTI-r sepsis or not (see Supplementary Material for details). Significant *p*-values are presented as bold, while the presence of * indicates that the multivariate analysis is also statistically significant for the biomarker. AKI: acute kidney injury; RTI-r: respiratory tract infection-related; sMer: soluble Mer.

The 7-day and 30-day mortality rates were 9.7% and 19.9%, respectively. At the univariate analysis, several variable results were statistically different between survivors and nonsurvivors, both at seven and 30 days. However, neither Gas6 nor sMer were significantly related to mortality at either timepoint (*p* > 0.05) (Table S2 and Figure 1a–d). At the multivariate analysis, the age, heart rate, respiratory rate, ratio between partial pressure of oxygen and fractional inspired oxygen (PaO_2_/FiO_2_) and Sepsis-related Organ Failure Assessment score were independent predictors of 30-day mortality (Table S3).

### 3.3. Acute Kidney Injury

AKI was present in 28% of patients (N. 249/890). Patients with AKI resulted in being more frequently affected by cardiovascular disease (CVD) and chronic kidney disease (CKD) and presented higher levels of severity scores compared to patients without AKI. Moreover, at ED admission they showed a lower mean arterial pressure (MAP) and a higher respiratory rate compared to other patients (*p* < 0.05) (Table S4).

The Gas6 concentration was significantly higher in AKI patients (34.8 [26.4–47.5] ng/mL) compared to non-AKI ones (29.8 [22.1–41.6] ng/mL; *p* < 0.001). Similarly, the sMer concentration was higher in AKI patients (9.8 [4.1–17.8] ng/mL) compared to non-AKI ones (7.9 [3.8–12.9] ng/mL, *p* = 0.005) (Table S4 and Figure 1e,f). The logistic regression model identified Gas6 (odds ratio (OR) 1.01, 95% Confidence Interval (CI) 1.00–1.02, *p* = 0.01), but not sMer, as independent predictors of AKI together with CKD, MAP, plasma lactate and white blood cells (Table S5).

### 3.4. Sepsis Related to Respiratory Tract Infection

RTI-r sepsis was present in 62.2% of patients (554/890), who resulted in being older and more frequently affected by chronic obstructive pulmonary disease and CVD, with a higher respiratory rate and lower pulse oxygen saturation (Table S6). The Gas6 plasma concentration was significantly lower in patients with RTI-r sepsis (29.8 [22.3–40.0] vs. 33.6 [24.8–46.8] ng/mL, *p* < 0.001), while sMer was not different in the two groups (8.3 [4.0–14.4] vs. 8.2 [3.9–14.8] ng/mL, *p* = 0.75) (Table S6 and Figure 1g,h). Gas6 was not an independent factor for RTI-r sepsis (Table S7). A subgroup analysis dividing RTI-r sepsis according to PaO_2_/FiO_2_ > or ≤300 is detailed in Table S8.

### 3.5. Coaguopathy

A total of 127 (14.3%) patients were taking anticoagulant therapy of any kind. No significant difference was observed in Gas6 plasmatic levels between patients taking anticoagulant therapy and those who did not (29.3 [22.5–42.1] ng/mL vs. 31.4 [23.2–44.1] ng/mL, respectively; *p* = 0.19). Analogously, sMer plasmatic levels were not different between patients taking anticoagulants and the other ones (8.2 [4.2–13.1] ng/mL vs. 8.3 [3.7–14.4] ng/mL, respectively; *p* = 0.76). To avoid possible confounders, when we investigated the following different aspects of coagulopathy, we excluded the 127 patients taking anticoagulants.

**Figure 2:**
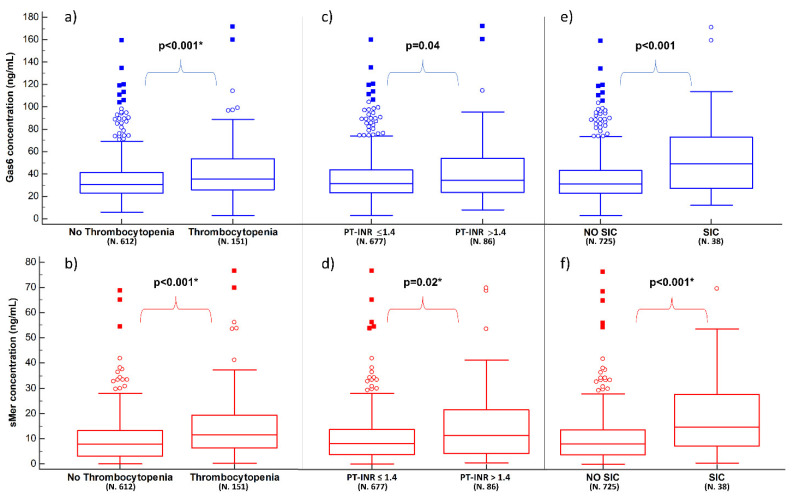
Plasma concentrations of Gas6 (blue boxes) and sMer (red boxes) according to thrombocytopenia, PT-INR derangement and SIC. Panels (**a**,**b**) show the results in relation to having thrombocytopenia or not; panels (**c**,**d**) show the results in relation to having PT-INR> or ≤1.4; panels (**e**,**f**) show the results in relation to having SIC or not (see Supplementary Material for details). Significant *p*-values are presented as bold, while the presence of * indicates that the multivariate analysis is also statistically significant for the biomarker. SIC: sepsis-induced coagulopathy.

Thrombocytopenia (platelets count < 150,000/mm^3^) was present in 19.8% (151/763) of patients. Patients with thrombocytopenia showed higher median values of both Gas6 (35.6 [25.9–53.8] vs. 30.7 [22.7–41.6] ng/mL; *p* < 0.001) and sMer (11.4 [6.4–19.2] ng/mL vs. 7.8 [3.1–13.3] ng/mL, *p* < 0.001) when compared to patients with a normal platelets count (Table S9 and Figure 2a,b). In the multivariate analysis, both Gas6 and sMer emerged as independent predictors of thrombocytopenia with ORs of 1.01 [95% CI 1.00–1.02] (*p* = 0.02) and 1.04 [95% CI 1.02–1.06] (*p* < 0.001), respectively (Table S10).

Derangement of PT-INR (>1.4) was present in 11.3% (86/763) of the patients. Patients with PT-INR > 1.4 showed a higher median concentration of both Gas6 (33.9 [23.5–53.5] vs. 31.1 [23.0–43.3] ng/mL, *p* = 0.04) and sMer (11.2 [4.0–21.6] vs. 8.1 [3.7–13.7] ng/mL, *p* = 0.02) when compared to the ones with PT-INR ≤ 1.4 (Table S11 and Figure 2c,d). However, in the multivariate analysis, only sMer was confirmed to be an independent predictor of PT-INR > 1.4 (OR 1.03 [95% CI 1.00–1.05], *p* = 0.02), together with plasma lactate, MAP and hemoglobin (Table S12).

Lastly, SIC was found in about 5% (38/763) of the considered patients. Patients with SIC showed a higher median concentration of both biomarkers compared to patients without SIC (49.2 [27.3–73.4] ng/mL vs. 31.2 [22.8–43.3] ng/mL, respectively for Gas6; 14.8 [7.2–27.7] ng/mL vs. 8.1 [3.7–13.7] ng/mL, respectively for sMer; *p* < 0.001 for both) (Table S13 and Figure 2e,f). In the multivariate analysis, sMer, but not Gas6, resulted in being an independent predictor of SIC with an OR of 1.05 ([95% CI 1.02–1.07], *p* < 0.001) (Table S14).

## 4. Discussion

Early sepsis recognition is a cornerstone of the management of septic patients, as remarked by the latest Surviving Sepsis Campaign guidelines [7]. However, this goal remains challenging, since clinical manifestations of sepsis are nonspecific and often shaded, especially during the first phase of the process, as well as in the elderly. Both these conditions were frequent in our patients, enrolled at the first medical contact in the ED, with a median age of 80 years. As the last definition of sepsis was centered on the presence of organ damage [30], its early identification may be the winning strategy for optimizing risk stratification, thus recognizing patients at a higher risk of death or worsening of organ damage.

Mer is a membrane tyrosine kinase receptor that belongs to the TAM receptor family. These receptors have pleiotropic effects on inflammation and hemostasis together with their ligand Gas6 [31]. During inflammatory processes, the cleavage of the extracellular part of Mer by ADAM17 results in the release of sMer [32]. Two studies involving critically ill patients admitted to ICUs with sepsis or septic shock showed that Gas6 and Mer were associated with increased mortality [20,21]. However, in our cohort, the plasma concentrations of Gas6 and sMer were similar among survivors and nonsurvivors both at seven and 30 days from enrolment. The reasons for this discrepancy may be different: for instance, Guignant and colleagues [20] evaluated Mer expression on the surface of the immune cells and not the plasma concentration of its soluble form. Moreover, both studies involved ICU-admitted patients who may have faced a more severe and/or advanced condition (as suggested also by the higher mortality rates), while our measurements captured an earlier condition in the ED.

What is more interesting is the association of increased plasma levels of these biomarkers in patients with different types of organ damage. Our results show that both sMer and Gas6 concentrations were higher in patients with thrombocytopenia, PT-INR derangement and SIC. In particular, sMer showed the strongest association, as also confirmed in the multivariate analysis, for all these conditions. This is somehow expected, since it is well known that these proteins are expressed by platelets, endothelial cells and leukocytes [33,34] and that they participate in the regulation of the thrombotic response [35]. Since coagulopathy is an independent predictor of a poor outcome in sepsis [36,37], the identification by Iba and colleagues of items to define the SIC score [38] seems to be helpful in improving the prognostic value. Recently, four clinical phenotypes for sepsis have been described by Seymour and colleagues [39], and it is possible that SIC is one of the patterns of organ damage underlying one or more of these phenotypes. In this regard, our findings on the association between SIC and both sMer and Gas6 may provide additional information for understanding and approaching this condition.

AKI is very frequent (28% in our cohort) in patients with sepsis and septic shock [40], and we found higher plasma levels of Gas6 and sMer in these patients. Other authors reported a higher plasma and urinary concentration of soluble TAM receptors (Tyro 3, Axl, Mer) and of their ligand (protein S) in patients with diabetic nephropathy [41]. It has also been hypothesized that the Gas6/Axl axis is involved in the pathway that leads to kidney dysfunction by promoting epithelial-to-mesenchymal transition in renal tubular cells [42]. The current definition of AKI [43] presents several limitations and potential pitfalls related to the use of markers of kidney function like creatinine and urine output [44]. For this reason, it has recently been proposed that one use biomarkers of kidney stress and/or injury in addition to current KDIGO criteria to better stage and identify early forms of AKI [45]. In this regard, biomarker-guided treatment strategies to prevent AKI in patients undergoing surgery [46,47] and in patients with sepsis [48] have already been proposed. However, whether Gas6 and sMer could act as specific biomarkers of kidney injury still needs to be clarified.

Previous results from our group found that Gas6 concentrations were higher in patients with dyspnea related to heart failure or pulmonary/systemic infections when compared to other causes of dyspnea (i.e., pulmonary embolism) or healthy volunteers [49]. Even if our results showed a lower plasma concentration of Gas6 in patients with RTI-r sepsis, more sick patients with PaO_2_/FiO_2_ ≤ 300 had slightly higher levels of Gas6. In a mouse model of sepsis, treatment with rmGas6 was shown to reduce the production of serum organ damage markers and proinflammatory cytokines, thus resulting in the reduction of acute lung injury [50]. An in vitro study suggested a similar anti-inflammatory activity for Gas6 and Mer when monocytes-macrophage cells were stimulated by lipopolysaccharide [51]. Higher plasma levels of Gas6 were also found in critically ill patients with sepsis who developed lung injury [23]. It may be possible that the interaction between respiratory sepsis and Gas6 is present only in the most severe conditions.

### Limitations

First, this study is a secondary analysis of a sub-cohort of the Need-Speed trial, which was designed with an original and different purpose [17]. Second, some data for the biomarkers were missing, due to the unavailability of the samples, since all aliquots were used for the original study. However, this was an occurrence for very few patients (<4%). Third, the Need-Speed trial was conducted between 2013 and 2015 and used the SIRS [24] instead of the latest Sepsis-3 criteria [30], which would have led us to enroll a slightly different population. However, we focused our analysis on organ dysfunction/damage, which is one of the highlights of the latest criteria. Fourth, information regarding previous or ongoing treatments was not reported, except for the use of anticoagulant therapy. It is possible that other treatments may have an impact not only on the development of organ damage but also on the plasma concentrations of the studied biomarkers.

## 5. Conclusions

Baseline levels of sMer and Gas6 were not associated with 7- and 30-day mortality in patients with sepsis at ED presentation. However, sMer emerged as an independent predictor of thrombocytopenia, PT-INR derangement and SIC in septic patients who were not taking an anticoagulant. Additionally, Gas6 independently predicted thrombocytopenia and AKI.

## Figures and Tables

**Table 1:** Baseline characteristics of the 890 patients with sepsis.

**General Characteristics**	
Age (years)	80 (72–87)
Sex, male/female	477 (54%)/413 (46%)
Body mass index	24.2 (21.7–27.3)
**Comorbidities**	
Arterial hypertension	399 (45%)
Cardiovascular disease	479 (54%)
Chronic obstructive pulmonary disease	231 (26%)
Chronic kidney disease	202 (23%)
Diabetes	243 (27%)
**Clinical parameters**	
Systolic blood pressure (mmHg)	120 (110–137)
Diastolic blood pressure (mmHg)	70 (60–80)
Mean arterial pressure (mmHg)	87 (77–97)
Heart rate (bpm)	100 (90–110)
Respiratory rate (bpm)	24 (20–28)
Pulse oxygen saturation (%)	94 (92–96)
Glasgow coma scale	15 (15–15)
Temperature (°C)	37.7 (36.6–38.2)
**Laboratory data**	
White blood cells (×10^3^/mm^3^)	12.9 (9.3–17.0)
Hemoglobin (g/dL)	12.2 (10.8–13.5)
Platelets (×10^3^/mm^3^)	221 (157–300)
Glucose (mg/dL)	131 (109–167)
Creatinine (mg/dL)	1.08 (0.83–1.67)
Total bilirubin (mg/dL)	0.91 (0.66–1.43)
PT-INR	1.19 (1.10–1.36)
aPTT (seconds)	30 (28–34)
C-reactive protein (mg/dL)	10.11 (3.42–18.62)
Lactate (mmol/L)	1.54 (1.09–2.22)
PaO_2_/FiO_2_	286 (230–346)
**Biomarkers**	
Gas6 (ng/mL)	31.1 (23.2–43.5)
sMer (ng/mL)	8.3 (4.0–14.4)
**Scores**	
SOFA	3 (1–4)
APACHE II	13 (10–16)
SAPS II	36 (30–42)
**Mortality**	
7-day	87 (9.7%)
30-day	177 (19.9%)

APACHE: Acute Physiologic Assessment and Chronic Health Evaluation [27]; aPTT: activated partial thromboplastin time; PaO_2_/FiO_2_: ratio between partial pressure of oxygen and fractional inspired oxygen; PT-INR: prothrombin time-international normalized ratio; SAPS: Simplified Acute Physiology Score [28]; SOFA: Sepsis-related Organ Failure Assessment [29].

## Data Availability

Individual de-identified participant data are available from the corresponding author on reasonable request.

## Supplementary Materials

The following supporting information can be downloaded at https://www.mdpi.com/article/10.3390/biomedicines10020198/s1.
