# Supplementary material for: Are Baseline Levels of Gas6 and Soluble Mer Predictors of Mortality and Organ Damage in Patients with Sepsis? The Need-Speed Trial Database
Source: Biomedicines. 2022 Jan 18;10(2):198. doi: 10.3390/biomedicines10020198 (PMC8869255; doi:10.3390/biomedicines10020198)
Supplement: Supplementary file 1 [file biomedicines-10-00198-s001.zip › biomedicines-1535952-supplementary.pdf]

SUPPLEMENTARY MATERIAL

Are baseline levels of Gas6 and soluble Mer predictors of mortality and organ damage in patients with sepsis? The Need-Speed trial database

Francesco Gavelli [1,2], Luca Molinari* [1,2], Marco Baldrighi [1,2], Livia Salmi [1], Filippo Mearelli [3], Nicola Fiotti [3], Filippo Patrucco [1], Chiara Airoldi [1], Mattia Bellan [1], Pier Paolo Sainaghi [1], Salvatore Di Somma [4], Enrico Lupia [5], Efrem Colonetti [6,7], Maria Lorenza Muiesan [6], Gianni Biolo [3], Gian Carlo Avanzi [1,2], Luigi Mario Castello [1,8].

[1] Department of Translational Medicine; Università del Piemonte Orientale, Novara 28100, Italy.
[2] Emergency Medicine Department; Azienda Ospedaliero-Universitaria "Maggiore della Carità", Novara 28100, Italy.
[3] Unit of Internal Medicine; Department of Medicine, Surgery and Health Sciences, University of Trieste, Trieste 34129, Italy.
[4] Unit of Emergency Medicine; Department of Medical Surgical Sciences and Translational medicine, University "Sapienza" of Rome, Rome 00189, Italy.
[5] Unit of Emergency Medicine; Department of Medical Sciences, University of Turin, Torino 10126, Italy.
[6] Unit of Internal Medicine; Department of Clinical and Experimental Sciences, University of Brescia, Brescia 25121, Italy.
[7] SEUM 118; Azienda Ospedaliera Universitaria Integrata di Verona, Verona 37126, Italy.
[8] Unit of Internal Medicine; Azienda Ospedaliera SS. Antonio e Biagio e Cesare Arrigo, Alessandria 15121, Italy.

* Correspondence: luca.molinari@med.uniupo.it; Tel.: +39 03213733247 / +39 3401289119.

INDEX OF CONTENTS

MATERIALS AND METHODS – BIOMARKERS ANALYSIS 3

Samples collection 3

Gas6 3

sMer 3

MATERIALS AND METHODS – ORGAN DAMAGE ASSESSMENT 4

Acute Kidney Injury 4

Respiratory tract infection-related sepsis 4

Coagulopathy 4

BOX AND WHISKERS PLOTS 5

Outside values 5

Far out values 5

SUPPLEMENTARY TABLES 6

Table S1 – Scoring for the diagnosis of sepsis-induced coagulopathy (SIC) 6

Table S2 – Univariate analysis for mortality at 7 and 30 days 7

Table S3 – Cox proportional-hazard regression model for 30-day mortality 8

Table S4 – Univariate analysis for AKI 9

Table S5 – Logistic regression model for independent predictors of AKI 10

Table S6 – Univariate analysis for RTI-r sepsis 11

Table S7 – Logistic regression model for independent predictors of RTI-r sepsis 12

Table S8 – Univariate analysis according to PaO2/FiO2 > or ≤ 300 in patients with RTI-r sepsis 13

Table S9 – Univariate analysis for thrombocytopenia 14

Table S10 – Logistic regression model for independent predictors of thrombocytopenia 15

Table S11 – Univariate analysis for PT-INR >1.4 16

Table S12 – Logistic regression model for independent predictors of PT-INR >1.4 17

Table S13 – Univariate analysis for SIC 18

Table S14 – Logistic regression model for independent predictors of SIC 19

SUPPLEMENTARY FIGURES 20

Figure S1 – Study flow-chart of the selection of patients for our analysis 20

REFERENCES 21

MATERIALS AND METHODS – BIOMARKERS ANALYSIS

Samples collection

Blood samples for biomarkers analysis were collected and centrifuged within 24 hours from Emergency Department admission. The aliquots were then stored at -80°C in the local laboratory facility of each Unit and then sent to the main Unit of Investigation (Laboratory of Emergency Medicine, Department of Translational Medicine, Università del Piemonte Orientale, Novara, Italy) for the determination of the plasma concentrations of the biomarkers.

Gas6

Gas6 was measured with a sandwich ELISA developed and validated in our laboratory [1]. A 96-well plate was coated overnight with anti-Gas6 capture antibody (goat polyclonal affinity-purified IgG, catalog number AF885, R&D Systems, Minneapolis, MN, USA). The plasma samples were diluted 1:100, then human Gas6 was detected by a secondary biotinylated antibody (goat polyclonal IgG antigen affinity-purified, human Gas6 biotinylated antibody, catalog number BAF885, R&D Systems, Minneapolis, MN, USA), streptavidin–peroxidase conjugate (Sigma-Aldrich, Sant-Louis, MO, USA) and TMB (3,3',5,5'-tetramethylbenzidine). The reaction was blocked with sulfuric acid 2 N and absorbance detected at 450 nm.

sMer

sMer was measured with a commercial ELISA kit (DuoSet ELISA catalog number DY6488, R&D System, Minneapolis, MN, USA). Following the kit instructions, a 96-well plate was coated overnight with a mouse anti-human Mer capture antibody. The plasma samples were diluted 1:4. Human Mer was then detected by a biotinylated goat anti-human Mer detection antibody, streptavidin–peroxidase conjugate and TMB. The reaction was blocked with sulfuric acid 2 N and absorbance detected at 450 nm.

MATERIALS AND METHODS – ORGAN DAMAGE ASSESSMENT

Acute Kidney Injury

Acute kidney injury (AKI) was defined by an increase in serum creatinine of 0.3 mg/dL or ≥ to 1.5-fold from baseline within 48 hours, according to the Kidney Disease: Improving Global Outcome (KDIGO) criteria for AKI [2].

Respiratory tract infection-related sepsis

Sepsis related to a respiratory tract infection (RTI-r sepsis) was defined as the presence of sepsis related to a documented clinical or microbiological lung infection, together with pulmonary opacities not suggestive of pleural effusion, atelectasis, or nodules on chest X-ray. Subsequently, the severity of RTI-r sepsis was categorized according to the presence of an arterial oxygen tension over inspired oxygen fraction ratio ($PaO2/FiO2$) > or ≤300.

Coagulopathy

Coagulopathy was defined according to the criteria for sepsis-induced coagulopathy (SIC) published by Iba and colleagues [3]. The criteria are based on three items: prothrombin time-international normalized ratio (PT-INR), platelets count, and four-items SOFA score (respiratory, cardiovascular, hepatic, and renal SOFA). For each of these items, a score from 0 to 2 points is given. SIC is established when the total score is ≥ 4, provided that the total score of prothrombin time and coagulation exceeds 2 (Table S1) [3]. Other than SIC, we evaluated separately two main components of the SIC score: thrombocytopenia (defined as platelets count < $150000/mm^3$) and PT-INR derangement (defined as PT-INR > 1.4).

BOX AND WHISKERS PLOTS

Figure 1 and Figure 2 show box and whiskers plots of the plasma concentrations of Gas6 (blue boxes) and sMer (red boxes). The central box represents the values from the lower to upper quartile (25 to 75 percentile). The middle line represents the median. A line extends from the minimum to the maximum value, excluding "outside" and "far out" values which are displayed as separate points.

Outside values

An “outside” value is defined as a value that is smaller than the lower quartile minus 1.5 times the interquartile range, or larger than the upper quartile plus 1.5 times the interquartile range (empty round dots).

Far out values

A “far out” value is defined as a value that is smaller than the lower quartile minus 3 times the interquartile range, or larger than the upper quartile plus 3 times the interquartile range (full square dots).

SUPPLEMENTARY TABLES

Table S1 – Scoring for the diagnosis of sepsis-induced coagulopathy (SIC)

SIC is diagnosed when the total score is 4 or more with total score of PT-INR and PLTs exceeding 2.

**Variables**	**0 point**	**1 point**	**2 points**
**PT-INR**	≤ 1.2	> 1.2	> 1.4
**PLTs, x $10^3/mm^3$**	≥ 150	< 150	< 100
**SOFA four items[a]**	0	1	≥ 2

Adapted from "Iba T, Nisio MD, Levy JH, Kitamura N, Thachil J. New criteria for sepsis-induced coagulopathy (SIC) following the revised sepsis definition: a retrospective analysis of a nationwide survey. BMJ Open. 2017 Sep 27;7(9):e017046. doi: 10.1136/bmjopen-2017-017046. PMID: 28963294; PMCID: PMC5623518" [3].

[a]**SOFA four items** is the sum of respiratory SOFA, cardiovascular SOFA, hepatic SOFA, and renal SOFA [4].
PLTs: platelets; PT-INR: prothrombin time-international normalized ratio; SOFA: Sepsis-related Organ Failure Assessment.

Table S2 – Univariate analysis for mortality at 7 and 30 days

Main general, clinical and laboratory data of the 890 patients divided according to being alive or dead at 7 days or at 30 days.

Variables	Alive at 7 days (N. 803)	Dead at 7 days (N. 87)	p-value	Alive at 30 days (N. 713)	Dead at 30 days (N. 177)	p-value
**General characteristics**						
**Age**	80 (71- 86)	85 (78 – 90)	**<0.001**	79 (71 - 86)	85 (78 - 90)	**<0.001**
**Sex, Female**	367 (46%)	46 (53%)	0.25	320 (45%)	93 (53%)	0.08
**BMI**	24.2 (21.7 - 27.3)	23.9 (21.3 - 27.3)	0.62	24.3 (21.9 - 27.4)	24.0 (20.9 - 26.4)	0.25
**Comorbidities**						
**AHT**	379 (47.2%)	20 (23.0%)	**<0.001**	333 (46.7%)	66 (37.2%)	**0.03**
**CVD**	427 (52.8%)	52 (60.0%)	0.29	361 (50.6%)	118 (66.7%)	**<0.001**
**COPD**	216 (26.9%)	15 (17.2%)	0.07	191 (26.8%)	40 (22.6%)	0.30
**CKD**	184 (22.9%)	18 (20.7%)	0.74	153 (21.5%)	49 (27.7%)	0.10
**Diabetes**	228 (28.4%)	15 (17.2%)	**0.04**	210 (29.5%)	33 (18.6%)	**0.005**
**Clinical parameters**						
**PAS, mmHg**	120 (110 - 140)	110 (90 - 120)	**<0.001**	120 (110 – 140)	110 (99 – 130)	**<0.001**
**PAD, mmHg**	70 (60 - 80)	60 (60 - 70)	**<0.001**	70 (60 – 80)	65 (60 – 70)	**<0.001**
**MAP, mmHg**	87 (77 - 97)	77 (70 - 90)	**<0.001**	87 (78 – 97)	81 (70 – 93)	**<0.001**
**HR, bpm**	99 (90 - 110)	110 (97 - 120)	**<0.001**	99 (90 – 110)	101 (95 – 115)	**<0.001**
**RR, bpm**	24 (20 - 26)	28 (24 - 32)	**<0.001**	23 (20 – 26)	26 (22 – 32)	**<0.001**
**POS, %**	94 (92 - 96)	93 (88 - 95)	**<0.001**	94 (92 – 96)	93 (90 – 96)	**<0.001**
**GCS**	15 (15 - 15)	14 (10 - 15)	**<0.001**	15 (15 – 15)	15 (12 – 15)	**<0.001**
**Temperature, °C**	37.8 (36.6 - 38.2)	37.2 (36.2 - 38.2)	0.11	37.9 (36.8 – 38.2)	37.1 (36.0 – 38.0)	**<0.001**
**Laboratory data**						
**WBC, x10^3/mm^3**	12.9 (9.4 – 16.9)	13.4 (9.5 – 18.9)	0.14	12.6 (9.2 – 16.8)	13.6 (9.8 – 18.3)	**0.03**
**Hb, g/dL**	12.2 (10.8 - 13.5)	12.0 (10.1 – 13.0)	0.19	12.2 (11.0 – 13.5)	12.0 (10.0 – 13.2)	**0.01**
**PLTs, x10^3/mm^3**	218 (158 - 294)	246 (155 – 330)	0.30	215 (156 – 293)	244 (167 – 320)	0.08
**Glucose, mg/dL**	130 (108 - 165)	135 (111 - 196)	0.28	130 (108 – 164)	136 (109 – 170)	0.33
**Creatinine, mg/dL**	1.07 (0.83 - 1.60)	1.31 (0.90 - 2.40)	**0.008**	1.06 (0.83 – 1.57)	1.26 (0.85 – 2.06)	**0.004**
**Bilirubin, mg/dL**	0.91 (0.66 - 1.40)	0.97 (0.70 - 1.86)	0.20	0.9 (0.66 – 1.38)	0.98 (0.66 – 1.56)	0.24
**PT-INR**	1.18 (1.09 - 1.35)	1.29 (1.18 - 1.38)	**<0.001**	1.17 (1.09 – 1.33)	1.26 (1.16 – 1.40)	**<0.001**
**aPTT, second**	30 (28 - 34)	31 (27 - 37)	0.52	30 (28 – 34)	30 (27 – 34)	0.68
**CRP, mg/dL**	9.6 (3.3 - 18.3)	13.3 (7.6 - 21.1)	**0.012**	9.3 (3.0 – 18.1)	12.9 (7.1 – 21.1)	**<0.001**
**Lactate, mmol/L**	1.50 (1.09 - 2.14)	2.31 (1.33 - 3.57)	**<0.001**	1.47 (1.06 – 2.10)	1.94 (1.30 – 3.02)	**<0.001**
**PaO_2/FiO_2**	291 (243 - 350)	212 (151 - 270)	**<0.001**	298 (248 – 357)	238 (160 – 295)	**<0.001**
**Biomarkers**						
**Gas6, ng/mL**	31.0 (23.2 - 43.2)	32.2 (23.1 - 47.1)	0.34	31.3 (23.3 – 43.4)	30.5 (22.6 – 44.2)	0.82
**sMer, ng/mL**	8.2 (3.7 - 13.8)	8.7 (4.6 - 16.9)	0.27	8.2 (3.6 – 13.8)	8.47 (4.4 – 15.5)	0.61
**Scores**						
**SOFA**	2 (1 - 4)	5 (3 - 7)	**<0.001**	2 (1 – 4)	4 (3 – 6)	**<0.001**
**APACHE II**	12 (9 - 15)	17 (14 - 22)	**<0.001**	12 (9 – 15)	15 (12 – 21)	**<0.001**
**SAPS II**	36 (30 - 40)	43 (38 - 50)	**<0.001**	35 (30 – 39)	42 (37 – 49)	**<0.001**

Continuous variables are presented as medians and interquartile range, categorical variables are presented as frequencies (%). **Bold** values indicate statistical significance according to $p<0.05$.

AHT: arterial hypertension; aPTT: activated partial thromboplastin time; BMI: body mass index; CKD: chronic kidney disease; COPD: chronic obstructive pulmonary disease; CRP: C-reactive protein; CVD: cardio-vascular disease; DBP: diastolic blood pressure; GCS: Glasgow Coma Scale; Hb: hemoglobin; HR: heart rate; bpm: beats/breaths per minutes; MAP: mean arterial pressure; PaO2/FiO2: ratio between partial pressure of oxygen and fractional inspired oxygen; PLTs: platelets; POS: peripheral oxygen saturation; PT-INR: prothrombin time-international normalized ratio; RR: respiratory rate; SAPS: Simplified Acute Physiology Score; sMer: soluble Mer; SOFA: Sepsis-related Organ Failure Assessment; SPB: systolic blood pressure; WBC: white blood cells.

Table S3 – Cox proportional-hazard regression model for 30-day mortality

Multivariate analysis performed to identify independent predictors of 30-day mortality.

Variables	Hazard Ratio	95% CI	p-value
**Age**	1.04	1.02 - 1.06	<0.001
**HR**	1.02	1.01 - 1.03	0.003
**RR**	1.04	1.01 - 1.07	0.02
**Temperature**	0.80	0.67 - 0.96	0.01
**Hb**	0.87	0.79 - 0.96	0.005
**PaO_2/FiO_2**	0.996	0.994- 0.999	0.01
**SOFA**	1.22	1.13 - 1.33	<0.001

CI: confidence interval; Hb: hemoglobin; HR: heart rate; PaO_2/FiO_2: ratio between partial pressure of oxygen and fractional inspired oxygen; RR: respiratory rate; SOFA: Sepsis-related Organ Failure Assessment.

Table S4 – Univariate analysis for AKI

This table represents the univariate analysis of several clinical and laboratory variables related to the presence or not of Acute Kidney Injury (AKI) according to KDIGO. In the second column are represented the data of 641 patients without AKI while in the third column the data of 249 patients with AKI.

Variables	Non-AKI (N. 641)	AKI (N. 249)	p-value
**General characteristics**			
**Age**	80 (71 - 86)	81 (75 - 88)	**0.002**
**Sex, M / F**	331 (52%) / 310 (48%)	146 (59%) / 103 (41%)	0.07
**BMI**	24.2 (21.1 – 27.3)	24.2 (22.0 – 26.7)	0.68
**Comorbidities**			
**AHT**	286 (44.6%)	113 (45.4%)	0.90
**CVD**	327 (51.0%)	152 (61.0%)	**0.007**
**CKD**	107 (16.7%)	95 (38.2%)	**<0.001**
**Diabetes**	172 (26.8%)	71 (28.5%)	0.67
**Clinical parameters**			
**MAP, mmHg**	87 (80 – 97)	83 (71 – 93)	**<0.001**
**HR, bpm**	100 (90 - 110	100 (90 – 100)	0.96
**RR, bpm**	24 (20 – 26)	24 (20 – 30)	**0.005**
**POS, %**	94 (92 – 96)	94 (91 – 96)	0.19
**Laboratory data**			
**WBC, x10^3/mm^3**	12.4 (9.0 – 16.6)	13.9 (10.1 – 18.7)	**0.001**
**Hb, g/dL**	12,4 (11,0 - 13,5)	11,7 (10,2 - 13,3)	**<0.001**
**PLTs, x10^3/mm^3**	224 (164 - 305)	207 (148 - 287)	**0.01**
**Creatinine, mg/dL**	0.96 (0.75 - 1.19)	2.00 (1.54 - 2.78)	**<0.001**
**Lactate, mmol/L**	1.46 (1.07 – 2.07)	1.80 (1.20 – 2.89)	**<0.001**
**Biomarkers**			
**Gas6, ng/mL**	29.8 (22.1 - 41.6)	34.8 (26.4 - 47.5)	**<0.001**
**sMER, ng/mL**	7.9 (3.8 - 12.9)	9.8 (4.1 - 17.8)	**0.005**
**Scores**			
**SOFA**	2 (1- 3)	4 (3- 6)	**<0.001**
**APACHE II**	11 (9 - 14)	15 (13- 19)	**<0.001**
**SAPS II**	34 (29 - 39)	40 (35 - 46)	**<0.001**

Continuous variables are presented as medians and interquartile range, categorical variables are presented as frequencies (%). **Bold** values indicate statistical significance according to p<0.05.

AHT: arterial hypertension; AKI: Acute Kidney Injury; BMI: body mass index; CKD: chronic kidney disease; CVD: cardio-vascular disease; Hb: haemoglobin; HR: heart rate; bpm: beats/breaths per minutes; KDIGO: Kidney Disease: Improving Global Outcomes; MAP: mean arterial pressure; PLTs: platelets; POS: peripheral oxygen saturation; RR: respiratory rate; SAPS: Simplified Acute Physiology Score; sMer: soluble Mer; SOFA: Sepsis-related Organ Failure Assessment; WBC: white blood cells.

Table S5 – Logistic regression model for independent predictors of AKI

Variables	Odds ratio	95% CI	p-value
**CKD**	3.27	2.25-4.73	<0.001
**MAP**	0.985	0.973-0.996	0.01
**WBC**	1.00	1.00-1.01	0.002
**Lactate**	1.28	1.13-1.43	<0.001
**Gas6**	1.01	1.00-1.02	0.01

AKI: Acute Kidney Injury; CI: confidence interval; CKD: chronic kidney disease; MAP: mean arterial pressure, WBC: white blood cells.

Table S6 – Univariate analysis for RTI-r sepsis

This table represents the univariate analysis of several clinical and laboratory variables related to the presence or not of Respiratory Tract Infection related (RTI-r) sepsis. In the second column are represented the data of 336 patients without RTI-r sepsis while in the third column the data of 554 patients with RTI-r sepsis.

Variables	NO RTI-r sepsis (N. 336)	RTI-r sepsis (N. 554)	p-value
**General characteristics**			
**Age**	78 (69 - 85)	82 (74 - 87)	**<0.001**
**Sex, M / F**	180 (53.6%) / 156 (46.4%)	299 (54.0%) / 255 (46.0%)	0.91
**BMI**	24.2 (22.0 – 27.3)	24.2 (21.6 – 27.3)	0.82
**Comorbidities**			
**COPD**	41 (12.2%)	190 (34.3%)	**<0.001**
**AHT**	147 (43.8%)	252 (45.5%)	0.61
**CVD**	160 (47.6%)	319 (57.6%)	**0.004**
**CKD**	75 (22.3%)	127 (22.9%)	0.84
**Diabetes**	104 (30.9%)	139 (25.1%)	0.06
**Clinical parameters**			
**MAP, mmHg**	87 (77 – 93)	87 (77 – 97)	**0.01**
**HR, bpm**	100 (92 – 110)	100 (90-110)	0.41
**RR, bpm**	22 (18 - 26)	24 (21 - 28)	**<0.001**
**POS, %**	95 (93 - 97)	94 (90 - 96)	**<0.001**
**Laboratory data**			
**WBCs, x10^3/mm^3**	13.3 (9.5 – 17.5)	12.6 (9.2 – 16.7)	0.12
**PLTs, x 10^3/mm^3**	204 (151 – 283)	231 (166 – 315)	**0.002**
**Creatinine, mg/dL**	1.13 (0.86 – 1.90)	1.05 (0.81 – 1.58)	**0.004**
**Lactate mmol/L**	1.56 (1.09 - 2.33)	1.53 (1.09 - 2.20)	0.76
**PaO2/FiO2**	344 (298 - 391)	260 (211- 308)	**<0.001**
**Biomarkers**			
**Gas6, ng/mL**	33.6 (24.8 - 46.8)	29.8 (22.3 - 40.0)	**<0.001**
**sMER, ng/mL**	8.2 (3.9 - 14.8)	8.3 (4.0 - 14.4)	0.75
**Scores**			
**SOFA**	2 (1 - 4)	3 (2 - 4)	**0.04**
**APACHE II**	12 (9 - 15)	13 (10 - 16)	**0.01**
**SAPS II**	35 (29 - 40)	36 (30 - 42)	**0.002**

Continuous variables are presented as medians and interquartile range, categorical variables are presented as frequencies (%). **Bold** values indicate statistical significance according to $p<0.05$.

AHT: arterial hypertension; BMI: body mass index; bpm: beats/breaths per minutes; CKD: chronic kidney disease; COPD: chronic obstructive pulmonary disease; CVD: cardio-vascular disease; HR: heart rate; MAP: mean arterial pressure; PaO2/FiO2: ratio between partial pressure of oxygen and fractional inspired oxygen; PLTs: platelets; POS: peripheral oxygen saturation; RR: respiratory rate; RTI-r sepsis: sepsis related to respiratory tract infection; SAPS: Simplified Acute Physiology Score; sMer: soluble Mer; SOFA: Sepsis-related Organ Failure Assessment; WBCs: white blood cells.

Table S7 – Logistic regression model for independent predictors of RTI-r sepsis

Variables	Odds ratio	95% CI	p-value
**COPD**	2.27	1.42 - 3.63	<0.001
**RR**	1.04	1.00 - 1.08	0.048
**POS**	0.92	0.87 - 0.98	0.01
**PaO2/FiO2**	0.990	0.988 - 0.993	<0.001

CI: confidence interval; COPD: chronic obstructive pulmonary disease; RR: respiratory rate; POS: peripheral oxygen saturation; PaO2/FiO2: ratio between partial pressure of oxygen and fractional inspired oxygen.

Table S8 – Univariate analysis according to PaO2/FiO2 > or ≤ 300 in patients with RTI-r sepsis

This table represents the univariate analysis of several clinical and laboratory variables related to the presence RTI-r sepsis (N. 554) with PaO2/FiO2 > or ≤ 300. In the second column are represented the data of 212 patients with RTI-r sepsis with PaO2/FiO2 > 300 while in the third column the data of 342 patients with RTI-r sepsis with PaO2/FiO2 ≤ 300.

Variables	PaO2/FiO2 > 300 (N. 212)	PaO2/FiO2 ≤ 300 (N. 342)	p-value
**Age**	82 (74 - 88)	81 (73 - 87)	0.42
**COPD**	61 (28.8%)	129 (37.7%)	**0.03**
**MAP, mmHg**	88 (80 - 97)	87 (77 - 97)	0.14
**RR, bpm**	24 (20 - 28)	25 (22 - 30)	**0.003**
**POS, %**	95 (94 - 97)	92 (89 - 94)	**<0.001**
**Lactate, mmol/L**	1.43 (1.06 - 2.00)	1.63 (1.13 - 2.32)	**0.01**
**Biomarkers**			
**Gas6, ng/mL**	29.0 (21.0 - 36.8)	30.6 (23.0 - 42.5)	**0.048**
**sMER, ng/mL**	8.1 (3.7 - 14.9)	8.5 (4.1 - 13.6)	0.90
**Scores**			
**SOFA**	2 (1 - 3)	3 (2 - 5)	**<0.001**
**APACHE II**	11 (9 - 14)	13 (11 - 17)	**0.003**
**SAPS II**	34.5 (30 - 40)	38 (33 - 44)	**<0.001**

Continuous variables are presented as medians and interquartile range, categorical variables are presented as frequencies (%). **Bold** values indicate statistical significance according to $p<0.05$.
bpm: beats/breaths per minutes; COPD: chronic obstructive pulmonary disease; MAP: mean arterial pressure; POS: peripheral oxygen saturation; RR: respiratory rate; RTI-r sepsis: respiratory tract infection related sepsis; SAPS: Simplified Acute Physiology Score; sMer: soluble Mer; SOFA: Sepsis-related Organ Failure Assessment.

Table S9 – Univariate analysis for thrombocytopenia

This table represents the univariate analysis of several clinical and laboratory variables related to the absence or presence of thrombocytopenia according to a platelets value ≥ or < 150000/mm^3. In the second column are represented the data of 612 patients without thrombocytopenia while in the third column the data of 151 patients with thrombocytopenia. Patients taking any kind of anticoagulant therapy (N. 127) were excluded from this analysis.

Variables	NO Thrombocytopenia (N. 612)	Thrombocytopenia (N. 151)	p-value
**General characteristics**			
**Age**	81 (72 - 87)	78 (66 - 85)	**0.005**
**Sex, M / F**	310 (51%) / 302 (49%)	93 (62%) / 58 (38%)	**0.02**
**Comorbidities**			
**AHT**	275 (44.9%)	54 (35.8%)	**0.04**
**CVD**	330 (53.9%)	61 (40.4%)	**0.003**
**CKD**	136 (22.2%)	36 (23.8%)	0.67
**Diabetes**	165 (27.0%)	37 (24.5%)	0.54
**Clinical parameters**			
**MAP, mmHg**	87 (77 – 97)	86 (73 - 95)	**0.02**
**HR, bpm**	100 (90 – 110)	100 (90 - 110)	0.83
**RR, bpm**	24 (20 - 28)	22 (20 – 26)	0.07
**POS, %**	94 (92 – 96)	94 (92 – 96)	0.89
**Temperature, °C**	37,6 (36,5 - 38,2)	38,0 (37,0 - 38,6)	**0.002**
**Laboratory data**			
**WBCs, x10^3/mm^3**	13.5 (10.1 – 17.4)	10.4 6.5 - 14.8)	**<0.001**
**Hb, g/dL**	12.2 (11,0 - 13,5)	12,2 (10,6 - 13,5)	0.28
**PT-INR**	1.16 (1.08 - 1.26)	1.22 (1.13 - 1.35)	**<0.001**
**Lactate, mmol/L**	1.48 (1.03 - 2.16)	1.90 (1.30 - 2.79)	**<0.001**
**Biomarkers**			
**Gas6, ng/mL**	30.7 (22.7 - 41.6)	35.6 (25.9 - 53.8)	**<0.001**
**sMER, ng/mL**	7.8 (3.1 - 13.3)	11.4 (6.4 - 19.2)	**<0.001**
**Scores**			
**SOFA**	2 (1 - 4)	5 (3 - 6)	**<0.001**
**APACHE II**	12 (10 - 16)	13 (10 - 17)	0.27
**SAPS II**	36 (30 - 41)	36 (30 - 43)	0.13

Continuous variables are presented as medians and interquartile range; categorical variables are presented as frequencies (%). **Bold** values indicate statistical significance according to p<0.05.

AHT: arterial hypertension; CVD: cardio-vascular disease; CKD: chronic kidney disease; MAP: mean arterial pressure; HR: heart rate; bpm: beats/breaths per minutes; RR: respiratory rate; POS: peripheral oxygen saturation; WBCs: white blood cells, Hb: hemoglobin; PT-INR: prothrombin time-international normalized ratio; sMer: soluble Mer; SOFA: Sepsis-related Organ Failure Assessment; SAPS: Simplified Acute Physiology Score.

Table S10 – Logistic regression model for independent predictors of thrombocytopenia

Variables	Odds ratio	95% CI	p-value
**Sex, Female**	0.62	0.40 – 0.96	0.03
**Temperature**	1.26	1.04 – 1.52	0.02
**WBCs**	0.99	0.99 – 1.00	<0.001
**Gas6**	1.01	1.00 – 1.02	0.02
**sMER**	1.04	1.02 – 1.06	<0.001

CI: confidence interval; WBCs: white blood cells; sMer: soluble Mer.

Table S11 – Univariate analysis for PT-INR >1.4

This table represents the univariate analysis of several clinical and laboratory variables related to the presence or absence of PT-INR derangement defined as PT-INR > or ≤ 1.4. In the second column are represented the data of 677 patients with a normal PT-INR value while in the third column the data of 86 patients with an increase PT-INR (>1.4). Patients taking any kind of anticoagulant therapy (N. 127) were excluded from this analysis.

Variables	PT-INR ≤ 1.4 (N. 677)	PT-INR >1.4 (N. 86)	p-value
**General characteristics**			
**Age**	80 (71 - 87)	79 (70 - 88)	0.91
**Sex, M / F**	353 / 324	50 / 36	0.35
**Comorbidities**			
**AHT**	303	26	**0.01**
**CVD**	342	49	0.26
**CKD**	155	17	0.51
**Diabetes**	178	24	0.75
**Clinical parameters**			
**MAP, mmHg**	87	80	**<0.001**
**HR, bpm**	100	100	0.63
**RR, bpm**	24	24	0.06
**POS, %**	94	94	0.69
**Laboratory data**			
**WBCs, x10^3/mm^3**	13.0	13.2	0.67
**Hb, g/dL**	12.4	11.6	**<0.001**
**PLTs, x 10^3/mm^3**	224 (166- 302)	178 (123 - 289)	**0.001**
**Lactate, mmol/L**	1.47 (1.06 - 2.12)	2.22 (1.51 - 3.54)	**<0.001**
**Biomarkers**			
**Gas6, ng/mL**	31.1 (23.0 - 43.3)	33.9 (23.5 - 53.5)	**0.04**
**sMER, ng/mL**	8.1 (3.7 - 13.7)	11.2 (4.0 - 21.6)	**0.02**
**Scores**			
**SOFA**	3 (1 - 4)	4 (3 - 6)	**<0.001**
**APACHE II**	12 (9 - 15)	16 (13 - 18)	**<0.001**
**SAPS II**	36 (30 - 41)	39 (34 - 46)	**<0.001**

Continuous variables are presented as medians and interquartile range; categorical variables are presented as frequencies (%). **Bold** values indicate statistical significance according to p<0.05.

PT-INR: prothrombin time-international normalized ratio; AHT: arterial hypertension; CVD: cardio-vascular disease; CKD: chronic kidney disease; MAP: mean arterial pressure; HR: heart rate; bpm: beats/breaths per minutes; RR: respiratory rate; POS: peripheral oxygen saturation; WBCs: white blood cells, Hb: haemoglobin; PLTs: platelets; sMer: soluble Mer; SOFA: Sepsis-related Organ Failure Assessment; SAPS: Simplified Acute Physiology Score.

Table S12 – Logistic regression model for independent predictors of PT-INR >1.4

Variables	Odds ratio	95% CI	p-value
**MAP**	0.98	0.96 - 0.99	0.046
**Hb**	0.79	0.69 - 0.90	<0.001
**Lactate**	1.28	1.12 - 1.45	<0.001
**sMER**	1.03	1.00 - 1.05	0.02

CI: confidence interval; MAP: mean arterial pressure; Hb: haemoglobin; sMer: soluble Mer.

Table S13 – Univariate analysis for SIC

This table represents the univariate analysis of several clinical and laboratory variables related to the absence or presence of SIC. In the second column are represented the data of 725 patients without SIC while in the third column the data of 38 patients with SIC. Patients taking any kind of anticoagulant therapy (N. 127) were excluded from this analysis.

Variables	NO SIC (N. 725)	SIC (N. 38)	p-value
**General characteristics**			
**Age**	81 (72 - 87)	77 (66 - 86)	0.21
**Sex, M / F**	378 (52%) / 347 (48%)	25 (66%)/ 13 (34%)	0.10
**Comorbidities**			
**AHT**	323 (44.6%)	6 (15.8%)	**<0.001**
**CVD**	375 (51.7%)	16 (42.1%)	0.25
**CKD**	166 (22.9%)	6 (15.8%)	0.31
**Diabetes**	192 (26.5%)	10 (26.3%)	0.98
**Clinical parameters**			
**MAP, mmHg**	87 (77 – 97)	77 (70 – 90)	**0.001**
**HR, bpm**	100 (90 - 110)	100 (93 – 110)	0.51
**RR, bpm**	24 (20 – 27)	24 (20 - 26)	0.64
**POS, %**	94 (92 - 96	94 (93 – 96)	0.66
**Laboratory data**			
**WBCs, x10^3/mm^3**	13.1 (9.6 – 17.1)	9.6 (6.2 – 16.0)	**0.005**
**Hb, g/dL**	12.2 (11 - 13.5)	12.0 (10.0 - 13.3)	0.19
**Lactate, mmol/L**	1.50 (1.07 - 2.18)	2.74 (1.80 - 4.20)	**<0.001**
**Biomarkers**			
**Gas6, ng/mL**	31.2 (22.8 - 43.3)	49.2 (27.3 - 73.4)	**<0.001**
**sMER, ng/mL**	8.1 (3.7 - 13.7)	14.8 (7.2 - 27.7)	**<0.001**
**Scores**			
**SOFA**	3 (1 - 4)	6 (5 - 8)	**<0.001**
**APACHE II**	12 (9 - 16)	16 (13 - 20)	**0.003**
**SAPS II**	36 (30 - 41)	42 (35 - 47)	**0.001**

Continuous variables are presented as medians and interquartile range, categorical variables are presented as frequencies (%). **Bold** values indicate statistical significance according to p<0.05.
AHT: arterial hypertension; bpm: beats/breaths per minutes; CKD: chronic kidney disease; CVD: cardio-vascular disease; Hb: haemoglobin; HR: heart rate; MAP: mean arterial pressure; PLTs: platelets; POS: peripheral oxygen saturation; RR: respiratory rate; SAPS: Simplified Acute Physiology Score; SIC: sepsis-induced coagulopathy; sMer: soluble Mer; SOFA: Sepsis-related Organ Failure Assessment; WBCs: white blood cells.

Table S14 – Logistic regression model for independent predictors of SIC

Variables	Odds ratio	95% CI	p-value
**AHT**	0.30	0.10 - 0.93	0.04
**MAP**	0.96	0.93 - 0.99	0.006
**WBC**	0.99	0.99 - 1.00	0.02
**Lactate**	1.17	1.02 - 1.35	0.03
**sMer**	1.05	1.02 - 1.07	<0.001

CI: confidence interval; AHT: arterial hypertension; MAP: mean arterial pressure; WBCs: white blood cells; sMer: soluble Mer.

SUPPLEMENTARY FIGURES

Figure S1 – Study flow-chart of the selection of patients for our analysis

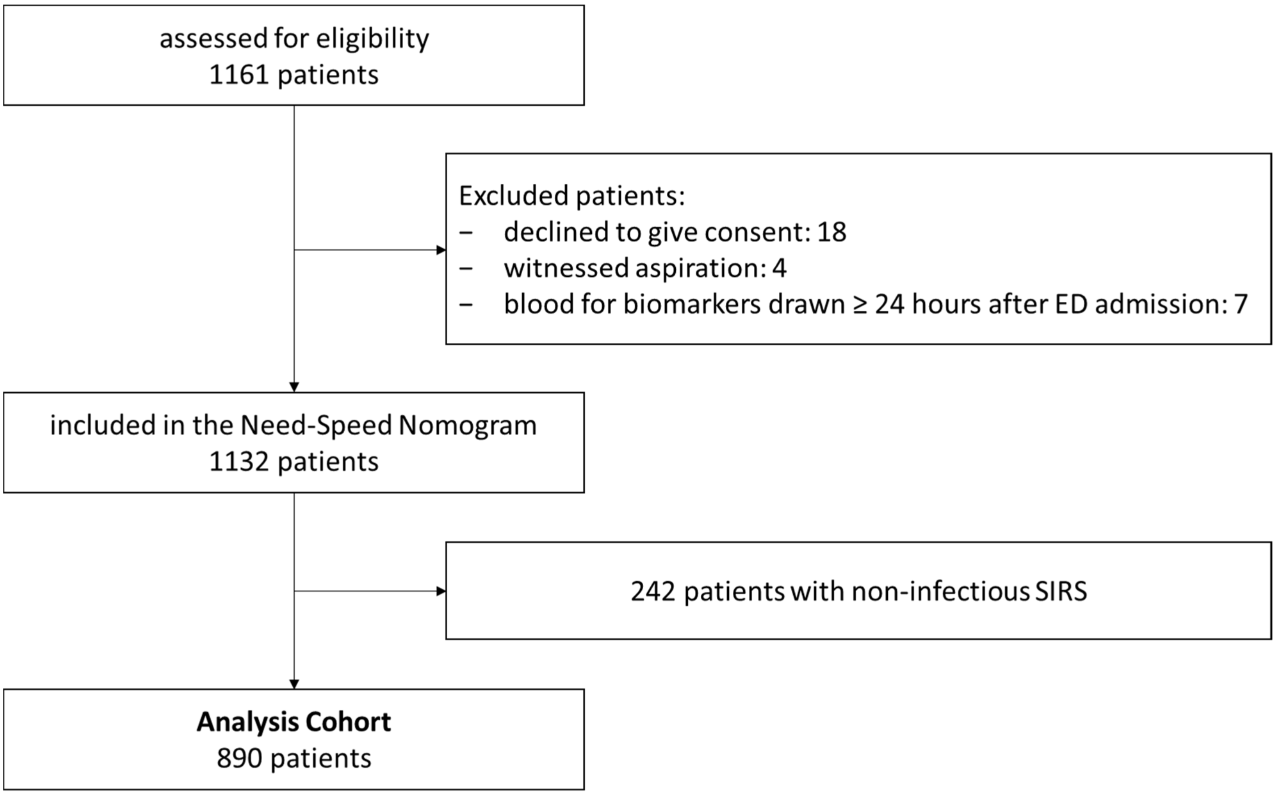

ED: emergency department; SIRS: systemic inflammatory response syndrome.

REFERENCES

1. Alciato, F.; Sainaghi, P.P.; Castello, L.; Bergamasco, L.; Carnieletto, S.; Avanzi, G.C. Development and validation of an ELISA method for detection of growth arrest specific 6 (GAS6) protein in human plasma. *J Immunoassay Immunochem* **2008**, *29*, 167-180, doi:10.1080/15321810801888480.
2. Kidney Disease: Improving Global Outcomes (KDIGO) Acute Kidney Injury Work Group. KDIGO Clinical Practice Guideline for Acute Kidney Injury. *Kidney Int Suppl* **2012**, *2*, 1-138, doi:10.1038/kisup.2012.1.
3. Iba, T.; Nisio, M.D.; Levy, J.H.; Kitamura, N.; Thachil, J. New criteria for sepsis-induced coagulopathy (SIC) following the revised sepsis definition: a retrospective analysis of a nationwide survey. *BMJ Open* **2017**, *7*, e017046, doi:10.1136/bmjopen-2017-017046.
4. Vincent, J.L.; Moreno, R.; Takala, J.; Willatts, S.; De Mendonça, A.; Bruining, H.; Reinhart, C.K.; Suter, P.M.; Thijs, L.G. The SOFA (Sepsis-related Organ Failure Assessment) score to describe organ dysfunction/failure. On behalf of the Working Group on Sepsis-Related Problems of the European Society of Intensive Care Medicine. *Intensive Care Med* **1996**, *22*, 707-710, doi:10.1007/BF01709751.
